# Physical functioning as a predictor of retirement: Has its importance changed over a thirty-year period in Sweden?

**DOI:** 10.1007/s10433-022-00725-y

**Published:** 2022-09-02

**Authors:** Harpa S. Eyjólfsdóttir, Neda Agahi, Johan Fritzell, Carin Lennartsson

**Affiliations:** 1 Aging Research Center, Karolinska Institutet and Stockholm University, Tomtebodavägen 18 A, 171 65 Solna, Sweden; 2 Swedish Institute for Social Research, Stockholm University, Stockholm, Sweden

**Keywords:** Retirement, Mobility limitations, Musculoskeletal pain, Gender, Cohort

## Abstract

Many countries, including Sweden, are implementing policies aimed at delaying retirement and encouraging older workers to remain on the labour market for longer. During recent decades, there have been several major reforms to the pension and social security systems in Sweden. Moreover, the nature of occupations has shifted towards more non-manual and sedentary activities, older women are today almost as active in the labour market as men in Sweden, and physical functioning has improved over time. In this study, we investigate whether the importance of physical functioning as a predictor for retirement has changed over time, for women and men, respectively. We used four waves of nationally representative data from The Swedish Level of Living Survey from 1981, 1991, 2000, and 2010, together with income register data. We found that greater severity of musculoskeletal pain and mobility limitations increased the likelihood of retirement in all waves. Results from logistic regression models with average marginal effects and predictive margins showed that there is a trend towards physical functioning becoming less important for retirement towards the end of the study period, especially for women, when controlling for occupational-based social class, age, adverse physical working conditions, and job demands. People, especially women, reporting impaired physical functioning did not retire to the same extent as in previous decades. This indicates that people stayed longer in the labour market despite impaired physical functioning, which may have repercussions on well-being and quality of life.

**Supplementary Information:**

The online version contains supplementary material available at 10.1007/s10433-022-00725-y.

## Introduction

We spend the majority of our adult life in the labour market, and for most, occupation is an important part of social identity (Ulfsdotter Eriksson and Linde 2014). Making the decision to retire is complex and influenced by institutional, individual, and labour market factors, which are not mutually exclusive (Börsch-Supan et al. 2009). Institutional factors refer to features of the pension system, such as eligibility age and the construction of the system. Individual factors include health, economic situation, family circumstance, and prospects of leisure activities. Poor physical, mental, and self-rated health increase the risk of unplanned and early retirement, disability pension, and unemployment (van Rijn et al. 2014). Labour market factors may be the occupational structure, working environment, and demand for certain skills (Sjögren Lindquist and Wadensjö 2009). Together, all these factors can influence the relationship between health and retirement, and the timing of retirement. In this study, we explore whether the importance of physical functioning as a predictor for retirement has changed over a three-decade period, using nationally representative data from Sweden.

Several changes have taken place in recent decades regarding the individual, the labour market, and society. The functional health status of older working-age adults has improved (Lindgren 2016), and the nature of occupations has gradually shifted from manual towards non-manual work (OECD 2013; Parker and Agahi 2013). Furthermore, in response to the ageing population most countries in Europe have carried out gradual and substantial pensions reforms over the last decades in order to delay effective exit from the labour market and thus enhance fiscal sustainability. These reforms consist of a range of measures, e.g. raising the eligibility age for pension, closing early exit pathways from the labour market and increases in the number of years of social security and tax contributions required to receive a (full) pension (Carone et al. 2016; European Commission 2021). Sweden has taken several measures in the past decades that have resulted in fewer opportunities for earlier retirement and stronger financial incentives for prolonging working life (European Commission 2021; Palme and Svensson 2010).

Taken together, it is therefore possible that the importance of determinants of retirement has changed during the past decades. We add to the literature by investigating whether the significance of physical functioning for retirement has changed over a three-decade period.

### Health before retirement

The role of health as a predictor of retirement has been recognized for a long time: studies in the 1970s found health-related work limitations to be predictive of early retirement (Bixby 1976), and since then, studies have consistently demonstrated the importance of health for retirement. A systematic review of 44 studies, including Swedish data, showed that self-rated health, mental health problems, chronic diseases, and musculoskeletal disorders all independently increased the risk of early retirement (van Rijn 2014).

Functional health status affects the timing of retirement; *mobility limitations* and *musculoskeletal pain* are two examples of such health issues. Mobility limitations have been found to be predictive of early work exit (Rice et al. 2011). Mobility limitations often represent a pre-clinical stage of disability and are associated with severe disability and high health care expenditures (Heiland et al. 2016; Musich et al. 2018). Studies have found that 10–20% of individuals aged 50–64 have mobility limitations (Gardener et al. 2006; Heiland et al. 2016; Nilsson et al. 2010) and that these limitations increase with age (Fritzell et al. 2007). Swedish studies have shown that women were more likely to report mobility limitations compared to men from the 1960s and onwards; however, both gender differences and the total proportion of older people with mobility limitations have decreased (Parker et al. 2008). It has also been established that there are socioeconomic differences in mobility limitations, where individuals of lower class experience earlier onset (Nilsson et al. 2011a, b) and greater risk of limitations (Avlund et al. 2004; Fritzell et al. 2007).

Musculoskeletal pain has consistently been identified as a predictor for early and disability retirement (Härkäpää 1992; Haukka et al. 2015; Kamaleri et al. 2009; Øverland et al. 2012; van Rijn 2014). A greater number of pain sites on the body have been associated with reduced self-reported physical and mental work ability, the anticipation that work ability will deteriorate, the feeling of being unable to continue working in one’s current job, and thoughts about retiring early (Miranda et al. 2010). The number of pain sites independently predicts disability pension retirement (Haukka et al. 2015; Kamaleri et al. 2009) and decreased functional ability (Kamaleri et al. 2008). Lower back pain, an important component of musculoskeletal pain, is the leading cause of years lived with disability in Europe, but the age-standardized point prevalence has decreased slightly from 1990 to 2017 (Wu et al. 2020). The prevalence of musculoskeletal pain increases with age (Rustøen et al. 2005), with female sex (Breivik et al. 2006; Schmitz and Lazarevič 2020; Wranker et al. 2016), and with lower socio-economic status (Dorner et al. 2018; Todd et al. 2019).

### The changing nature of occupations

Over the last few decades, the labour market has been subject to major changes driven by globalization and technological development. The nature of occupations has gradually shifted from factory work to service work (Baruch 2015; Parker and Agahi 2013), and from more physically strenuous to sedentary jobs (Johansson et al. 2018). Accordingly, the class structure of the labour market has changed in many respects since the 1980s. In Sweden, the share of unskilled manual workers has decreased from 32% in 1985 to 20% in 2015.

Advantages and disadvantages accumulate over the course of life; for example, there is evidence that exposure to adverse working conditions over a long period of time contributes to a decline in physical functioning (Mänty et al. 2016; Nilsen et al. 2017). Factors in the physical work environment, e.g. heavy lifting, repetitive movements, and loud noises, have shown a strong association with disability pension and early retirement (Sundstrup et al. 2018). Adverse working conditions are more predominant in manual occupations than non-manual. Older adults who have held manual occupations, have a low level of education or low income, are more likely to experience health problems and die at a younger age than older adults who have worked in non-manual occupations, have a higher level of education, or have higher incomes (Lennartsson et al. 2018; Rehnberg and Fritzell 2016). Thus, because of the changing nature of occupations, from manual to non-manual and from physically strenuous to sedentary, the importance of physical functioning as a predictor for retirement is likely to have diminished.

### Gender segregation on the labour market

The labour market is gender segregated: women are more likely to have lower-status jobs, work in the public sector in fields such as education, health care and caregiving, have lower wages, work part-time, and as a consequence, have lower pensions compared to men (Edge et al. 2017). Physical working conditions for many sectors have changed for the better, especially the male-dominated sectors. The change is, however, less notable in the typically female-dominated occupations such as caregiving, health care, and retail (Westerlund 2005). The typically female-dominated occupations involve frequent contact with people and meeting other people’s needs. Such tasks often have high psychosocial demands, they are more difficult to plan and control compared to tasks involving machinery (SOU 2015), and they are associated with higher stress levels (Anxo et al. 2014). Moreover, women are typically worse off regarding pain, multimorbidity, poor self-rated health, chronic health conditions, activity limitations, and depression (Schmitz and Lazarevič 2020). Women take more sick leave from work, report poorer health, and retire earlier compared to men (Anxo et al. 2014). Taken together, the female-dominated labour market has not seen as much change for the better, and pathways towards early retirement and disability pension are closing. This may lead to women working additional years despite health limitations.

### Pension reforms and labour force participation in Sweden

Sweden is an interesting case to study when it comes to determinants of retirement, partly because of the high employment rate among both women and men, and the high retirement age in an international comparison (OECD 2021), but also because Sweden has already implemented various pension reforms that other countries have on the agenda. Flexible retirement age was introduced in 2003 in Sweden, offering individuals increased freedom to choose when to retire. At the same time, other reforms have closed exit pathways out of the labour market, for example criteria for disability benefits have become stricter, the partial pension scheme has been abolished, and there are stronger financial incentives to stay in the labour market. Sweden has undertaken major policy reforms, and the spillover effects of such reforms have the potential to impact health. As many countries face major pension reforms in the near future, examples from Sweden are of more general interest. A description of major reforms in Sweden since the 1980s that possibly influenced retirement behaviour of the cohorts under study and thus are of significance to this study can be found in Supplementary material.

Since the 1960s, women’s labour force participation has increased dramatically in Sweden (König and Lindquist 2016). The employment rate among women aged 55–64 has increased from 54% in the early 1980s to almost 76% in 2019, while in the same period the employment rate among men has remained stable, just below 80% (Statistics Sweden 2020). The employment rate for people aged 55–64 is notably lower in Europe today: 62% for women and 72% for men on average. The average age for leaving the labour market, restricted to those in the labour force at the age of 50, was calculated at 63.3 years for women and 64.3 years for men in 2016. This reflects a substantial increase from 61 and 63.2 years in 1981 for women and men, respectively (Swedish Pensions Agency 2017). Over 80% of people retire at or before the age of 65 (Swedish Pensions Agency 2020).

### Current study

There is a bulk of evidence regarding the significance of good health and physical functioning for labour force participation. However, little is known about whether the importance of good physical functioning for employment has changed along with the shifting labour market and generally improved health among older people in the past decades. The main aim of this paper is therefore to explore whether the importance of physical functioning as a predictor for retirement has changed over a three-decade period, using nationally representative data from Sweden. Because of the gender segregation on the labour market, women’s worse physical functioning compared to men, stricter eligibility criteria for disability pension, and smaller technical advancements within female-dominated occupations, the second aim is to investigate whether developments differ for women and men.

We hypothesize that (1) physical functioning loses its predictive power for retirement during the study period and (2) the decrease in predictive power will be larger among women.

## Data and methods

### Material

The study is based on the Swedish Level of Living Surveys (LNU) from 1981, 1991, 2000 and 2010, and the linked Longitudinal integrated database for health insurance and labour market studies (LISA). LNU is a longitudinal study that started in 1968 and has since then been repeated at six-to-ten-year intervals. It is based on face-to-face interviews with a representative sample of the Swedish population in the age range of 18–75 years. Each consecutive wave also includes a new random sample of young people and new immigrants in addition to the original sample, thus ensuring that each wave remains representative of the total adult population from a cross-sectional perspective (Fritzell and Lundberg 2007). The response rate of LNU has varied between 82.4% (1981), 79.1% (1991), 76% (2000), and 61% (2010). LISA is a national income register covering all residents in Sweden older than 16 years (Ludvigsson et al. 2019). Data on retirement were obtained from LISA and linked to the survey data using personal identification numbers assigned to each Swedish resident.

**Fig. 1:**
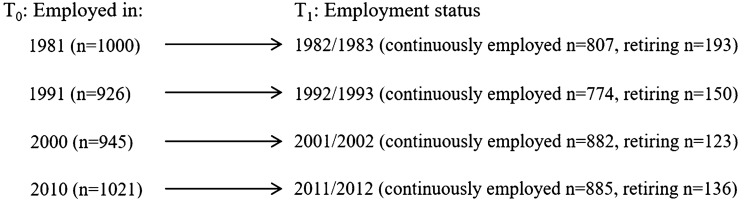
Sample construction. The figure shows the four waves of data collection: Baseline (*T*_0_) in 1981, 1991, 2000 and 2010 and two-year follow-up for each wave (*T*_1_). Sample size (n) at each baseline is indicated, and employment status at follow-up (continuously employed vs. retired)

This is a trend study with four baseline waves (*T*_0_ 1981, 1991, 2000 and 2010) with two-year follow-ups for each wave *T*_1_ (see Fig. 1). For example, participants in the *T*_0_ LNU 1981 wave are followed using income register data in the two consecutive calendar years *T*_1_ 1982–1983. All study participants who were aged 50–67 and employed (defined as more than 50% of their total individual annual income coming from employment) at baseline (*T*_0_) were included in the analysis, resulting in a total of 3890 cases over the study period (*T*_1_). Due to internal missing values, the sample size for the fully adjusted analysis with mobility limitations was *n* = 3862, and with musculoskeletal pain *n* = 3870.

### Measurements

#### Outcome

Employment status was identified through LISA, which includes total annual individual income from employment and self-employment, unemployment benefits, social security benefits including, for example, disability benefits, and annual pension income. The outcome variable in the analysis is *having retired within 2 years* (*T*_1_) after baseline interview (*T*_0_), thus separating those who retired from those who did not retire within two years of baseline interview. We are interested in exit from paid employment and not only the formal date of retirement (Hoffmann 2015), and for that reason we chose to set the retirement age as when pension income including disability benefits exceeds 50% of total annual income. This operationalization of retirement age has previously been described (Eyjólfsdóttir et al. 2021) and used in other studies of retirement using income registers, e.g. (Baumann et al. 2020; Eyjólfsdóttir et al. 2019; Svensson et al. 2015). A sensitivity analysis using different cut-offs for the definition of retirement (when income from pension exceeds 30%, 40%, 60%, and 70% of annual income) did not show differences against using the 50% cut-off, giving us confidence in the above-mentioned definition of retirement (Eyjólfsdóttir et al. 2021). As can be seen in Fig. 1, 193 individuals retired within two years of *T*_0_ in the 1981 wave, 150 in the 1991 wave, 123 in the 2000 wave, and 136 in the 2010 wave.

#### Exposures

Physical functioning was measured at *T*_0_ in the LNU survey: 1981, 1991, 2000, and 2010, respectively. *Mobility limitations* contained self-reported items on the ability to run 100 m, walk 100 m, and climb stairs without difficulty; response alternatives were ‘No’ and ‘Yes’. A categorical variable was created and answers coded as no limitations (0), one limitation (1), and two or more limitations (2). *Musculoskeletal pain* was measured with the question*: ‘Have you had any of the following illnesses or ailments during the past 12 months?’* followed by a list of health problems, three of which concern musculoskeletal pain: in the shoulders; in the back, hips, or sciatica; and in the hands, elbows, legs, or knees. For each item, the response alternatives were ‘No’, ‘Yes, mild problems’, and ‘Yes, severe problems’. Previous studies have shown that the number of pain sites independently predicts early retirement (Kamaleri et al. 2009; Miranda et al. 2010) and that functional limitations increase with the number of pain sites (Kamaleri et al. 2008). A categorical variable was created and answers coded as no pain site (0), one or two mild pain sites (1 “mild pain”), and three mild or at least one severe pain site or more (2 “severe pain”).

#### Covariates

*Occupational-based social class* is based on occupation and the typical skills required for the occupation. This measure follows the official Swedish socio-economic classification (Andersson et al. 1981), which in many ways corresponds to the internationally well-known Erikson–Goldthorpe (EGP) social class scheme (Erikson and Goldthorpe 1992). Occupational-based social class was collected at *T*_0_ in LNU survey and divided into four groups: (0) unskilled manual workers; (1) skilled manual workers, lower non-manuals with less than two years of post-comprehensive school education, small-scale farmers, and the self-employed without employees; (2) lower non-manuals with two years of post-comprehensive school education, farmers with extensive land and/or employees, and the self-employed with 1–19 employees; and finally (3) intermediate and higher non-manuals, academic professionals, and the self-employed with at least 20 employees. This categorization of occupational-based social class is common in the literature, see, e.g. (Baumann et al. 2020; Eyjólfsdóttir et al. 2019; Lennartsson et al. 2018). To adjust for changing working conditions over recent decades, we created a variable of *adverse physical working conditions* consisting of six items: sweating daily at work; the work being physically demanding in any way; working in uncomfortable physical positions; heavy lifting at work; being exposed to loud noise; and being exposed to gases. This variable ranged from 0 (not exposed) to 13 (exposed to all). To adjust for changes in *job demands* over the period, we created a variable consisting of two items that had simple yes or no response alternatives: psychosocially demanding work and hectic work. This variable was categorical, ranging from 0 to 2 where zero was having neither a demanding nor a hectic job.

### Statistical analysis

Logistic regression models were used to study the association between physical functioning and retirement for men and women separately by period. These models were adjusted for covariates (age, occupational-based social class, adverse physical working conditions, and job demands). Since there are differences between men and women in labour market involvement, we ran all analyses separately for men and women. Because of the unobserved heterogeneity that can vary across the compared samples, groups, or points in time, the more traditional odds ratios cannot be compared for models across groups, samples or time points (Mood 2010). Therefore, estimates are reported as average marginal effects (AMEs) with 95% confidence intervals (CI)s. AMEs facilitate interpretability and comparability across models for groups, samples or time points (Mood 2010). An AME can be interpreted as the average difference in the probability (0–1) of the outcome depending on the value of the independent variable, that is, it is the average change in probability of retirement (the outcome) when mobility limitations or musculoskeletal pain (the exposures) increase by one unit. Finally, we use predictive margins (PM) to illustrate the probability of retirement over the period by mobility limitations on the one hand, and musculoskeletal pain on the other. PMs are easier to interpret when presenting group differences than regression coefficients (Graubard and Korn 1999), especially in the presence of interaction terms. The PMs show the probability of retirement for all levels of the exposure variable while holding other variables in the model constant, while the AMEs use one level of the exposure variable as a reference category and show the discrete change from the reference category and if the difference is significant. All analyses were weighted to adjust for differences in response rates by some standard background variables, such as sex, age and education.

## Results

Table 1 shows the characteristics of the study population according to the year of baseline interview. In line with societal development over the last decades, we see that the average retirement age increased by two years and the proportion of intermediate and higher non-manual workers has increased from 1981 to 2010. Men report less exposure to adverse physical working conditions over the period, while there was no change among women. There was an increase in high job demands for women over the study period.

**Table 1:** Sample characteristics over period and gender, weighted percentages

	Period							
	1981		1991		2000		2010	
	Men (n = 552)	Women (n = 448)	Men (n = 483)	Women (n = 441)	Men (n = 493)	Women (n = 452)	Men (n = 535)	Women (n = 486)
*n*	54.8	45.2	51.6	48.4	51.4	48.6	51.3	48.7
Age at *T*_0_ (mean (range 50–67))	56.6	56.5	56.3	56.1	55.8	55.9	57.3	56.9
Retirement age (mean)	62.1	61.8	62.5	62.3	62.2	61.8	64.7	64.0
Employment status at *T*_1_								
Employed	83.9	77.0	81.5	84.2	87.0	87.2	87.4	87.0
Retired	16.1	23.0	18.5	15.8	13.0	12.8	12.6	13.0
Occupational social class at *T*_0_								
Unskilled manual workers	21.3	45.6	17.6	35.5	15.7	21.6	19.6	17.3
Skilled manual workers	36.1	27.6	35.9	35.8	35.6	37.7	36.6	41.4
Lower non-manuals	14.4	7.1	8.7	2.7	5.6	2.9	5.8	1.8
Intermediate and higher non-manuals	28.0	20.6	37.8	26.0	43.2	37.8	38.0	39.4
Adverse physical working conditions at *T*_0,_ [mean (range 0–13)]	3.8	2.0	3.2	2.0	2.8	1.9	2.9	2.0
Job demands at *T*_0_								
Low demands	35.8	39.8	32.8	26.1	32.5	27.5	29.0	22.9
Middle demands	33.3	37.2	34.3	35.6	33.3	35.3	35.1	31.1
High demands	30.9	23.0	32.9	38.3	34.2	37.1	35.9	46.0

The proportion of study participants reporting mobility limitations and musculoskeletal pain is displayed in Table 2. The prevalence of pain was generally higher than that of mobility limitations. Women displayed higher prevalence of both mobility limitations and severe pain than men throughout the entire study period. For both women and men, we see a decline in two or more mobility limitations, and a decline in severe pain among men. The prevalence of continuously employed women who report severe pain increased over the study period.

**Table 2:** Weighted proportion of study participants reporting mobility limitations and musculoskeletal pain at *T*_0_ by period, gender and employment status at *T*_1_

	No mobility limitations				1 mobility limitation				2+ mobility limitations			
	1981	1991	2000	2010	1981	1991	2000	2010	1981	1991	2000	2010
Total	76.1	78.9	81.5	81.0	10.0	11.6	10.9	11.8	13.9	9.5	7.6	7.2
Gender												
Men	78.5	81.5	87.6	86.4	9.5	9.5	8.1	8.4	12.0	9.0	4.4	5.2
Women	73.2	76.1	75.0	75.5	10.6	13.8	13.9	15.3	16.2	10.1	11.1	9.3
Employment status at *T*_1_												
Men, employed	80.1	85.0	90.6	88.4	9.7	7.7	7.2	7.6	10.2	7.3	2.1	4.0
Men, retired	70.3	66.1	67.1	72.4	8.5	17.3	13.8	13.9	21.2	16.7	19.1	13.7
Women, employed	78.7	79.7	77.1	77.3	9.6	12.8	14.0	13.5	11.7	7.5	8.9	9.3
Women, retired	54.9	56.4	60.8	63.2	13.8	19.5	13.2	27.5	31.4	24.1	26.1	9.3

	No pain				Mild pain				Severe pain			
	1981	1991	2000	2010	1981	1991	2000	2010	1981	1991	2000	2010
Total	46.9	39.4	37.0	44.9	25.1	30.6	32.8	28.9	28.0	30.0	30.2	26.2
Gender												
Men	50.2	42.6	41.6	50.2	24.3	30.8	33.9	28.1	25.5	26.7	24.5	21.8
Women	42.9	36.0	32.0	39.5	26.1	30.4	32.7	29.7	31.0	33.6	36.3	30.8
Employment status at *T*_1_												
Men, employed	51.7	42.7	42.4	49.2	24.2	31.0	34.2	29.7	24.1	26.3	23.4	21.2
Men, retired	42.2	42.2	36.2	56.9	25.0	29.6	31.5	17.4	32.9	28.2	32.4	26.7
Women, employed	44.1	35.1	33.7	38.8	28.9	32.7	32.1	30.5	27.1	32.3	34.2	30.7
Women, retired	38.8	41.2	20.6	43.8	16.9	18.1	28.9	24.5	44.3	40.7	50.5	31.6

Table 3 shows the AMEs of retiring within two years for men and women in each period, by mobility limitations on the one hand and musculoskeletal pain on the other, while adjusting for age, occupational-based social class, adverse physical working conditions, and job demands. For men in 1981, there were no differences in the probability of retiring within two years depending on the existence of or the number of mobility limitations. For men in 1991, there was a 19-percentage-point (AME 0.19, 95% CI 0.06, 0.33) higher probability of retiring within two years if reporting two or more mobility limitations compared to having no mobility limitations. In 2000, this association became stronger (AME 0.32, 95% CI 0.12, 0.52), but was lower again in 2010 at 12 percentage points. Women in 1981 with two or more mobility limitations had a 20-percentage-point higher probability of retiring within two years compared to women with no mobility limitations (AME 0.20, 95% CI 0.08, 0.32). This difference was smaller in 1991 (AME 0.10, 95% CI − 0.03, 0.23), then larger again in 2000, reaching statistical significance (AME 0.13, 95% CI 0.03, 0.23). The difference vanished in 2010; women reporting two or more mobility limitations did not differ from women reporting no mobility limitations in their probability of retiring within two years while controlling for age, occupational-based social class, adverse physical working conditions, and job demands. Full table of the findings from Table 3 can be found in Supplementary materials, Tables S1 and S2.

**Table 3:** Average marginal effects (AME) and 95% confidence intervals (CI) of retiring by mobility limitations and musculoskeletal pain for men and women in 1981, 1991, 2000, and 2010

	Men				Women			
	1981	1991	2000	2010	1981	1991	2000	2010
	AME (95% CI)	AME (95% CI)	AME (95% CI)	AME (95% CI)	AME (95% CI)	AME (95% CI))	AME (95% CI)	AME (95% CI)
Mobility limitations								
No limitations	Reference category				Reference category			
1 limitation	− 0.05 (− 0.12, 0.03)	0.07 (− 0.02, 0.16)	0.04 (− 0.04, 0.11)	− 0.01 (− 0.07, 0.04)	0.04 (− 0.04, 0.12)	0.07 (− 0.02, 0.15)	0.03 (− 0.05, 0.10)	0.05 (− 0.02, 0.11)
2+ limitations	0.02 (− 0.06, 0.09)	**0.19 (0.06, 0.33)**	**0.32 (0.12, 0.52)**	**0.12 (0.01, 0.24)**	**0.20 (0.08, 0.32)**	0.10 (− 0.03, 0.23)	**0.13 (0.03, 0.23)**	− 0.00 (− 0.08, 0.08)
Musculoskeletal pain								
No pain	Reference category				Reference category			
Mild pain	0.02 (− 0.04, 0.09)	− 0.05 (− 0.11, 0.02)	− 0.01 (− 0.06, 0.04)	*− 0.05 (− 0.09, 0.002)*	− 0.06 (− 0.12, 0.01)	**− 0.07 (− 0.13, − 0.01)**	0.03 (− 0.03, 0.08)	− 0.00 (− 0.05, 0.05)
Severe pain	*0.07 (− 0.00, 0.14)*	0.02 (− 0.06, 0.10)	0.05 (− 0.01, 0.12)	− 0.00 (− 0.06, 0.05)	**0.10 (0.02, 0.19)**	0.02 (− 0.05, 0.09)	**0.09 (0.03, 0.15)**	0.05 (− 0.01, 0.10)

Adjusted for age, occupational social class, adverse physical working conditions, and job demands. The results of the association between mobility limitations and retiring within two years are not adjusted for musculoskeletal pain, and vice versa

Bold values denote statistical significance at the *p* < 0.05 level*. *Italic values denote statistical significance at the *p* < 0.10 level

The results for musculoskeletal pain (Table 3) show a different trend for women and men. For women, we see that when comparing persons reporting severe pain to those reporting no pain, the probability of retirement in 1981 was 10 percentage points higher (AME 1981 0.10, 95% CI 0.02, 0.19). This difference became insignificant in 1991 and emerged again in 2000 with a 9-percentage-point higher probability of retirement. In 2010, the difference was smaller again but still statistically significant, when controlling for age, occupational-based social class, adverse physical working conditions, and job demands (AME 2010 0.05, 95% CI 0.01, 0.10). Among men, however, over the whole period, there was no statistically significant difference in the probability of retirement when comparing mild and severe pain to none, respectively.

**Fig. 2:**
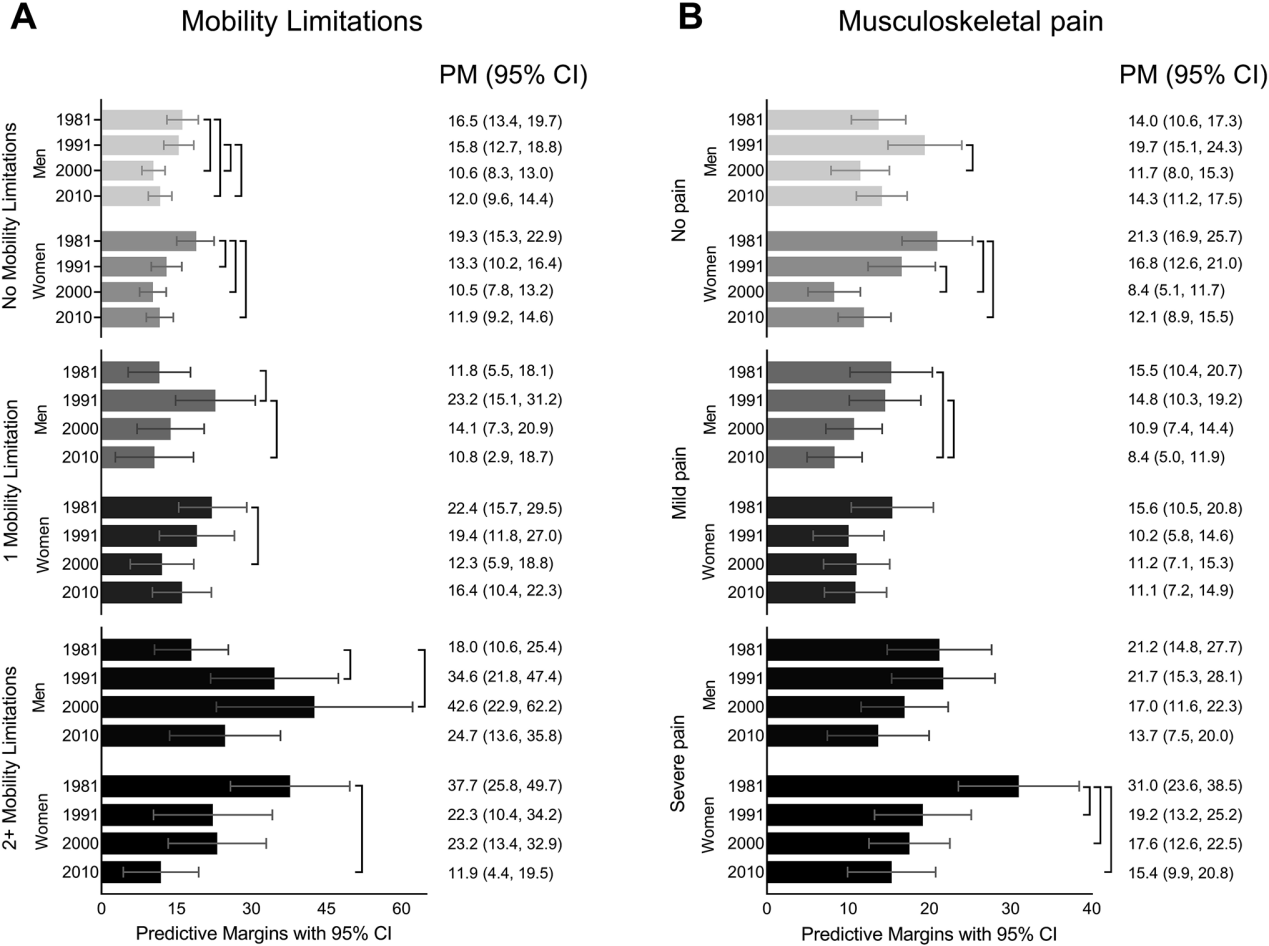
Predictive margins (PM) and 95% confidence interval (CI) of mobility limitations (**A**) and musculoskeletal pain (**B**) for men and women on the probability of retirement within two years, including a three-way interaction between gender, period, and the respective health outcome, while adjusting for age, occupational-based social class, adverse physical working conditions, and job demands.] indicates statistically significant difference at the *p* < 0.05 level (Cumming and Finch 2005)

Figure 2 shows the PM from a logistic regression of mobility limitations (A) and musculoskeletal pain (B) for men and women, including a three-way interaction between gender, period, and the respective health outcome on the probability of retirement within two years, while adjusting for age, occupational-based social class, adverse physical working conditions, and job demands. The PMs show the probability of retirement for all levels of the exposure variable while holding other variables in the model constant. Starting with mobility limitations (Fig. 2A): both among women and men with no mobility limitations the probability of retirement became lower during the period. The probability of retirement for women with no mobility limitations was significantly lowered from 1981 (19.3%) to 1991 (13.3%), 2000 (10.5%), and 2010 (11.9%). Men reporting two or more mobility limitations in 1981 had a relatively low probability of retirement (18%). The probability was higher in 1991 and also in 2000 at 42.6% (95% CI 22.9, 62.2). The probability of retirement was lower in 2010 at 24.7% (95% CI 0.13.6, 35.8). Women who reported two or more mobility limitations had a 38% probability of retirement in 1981, 22% probability in 1991 and 2000, and again lower in 2010 at 12% probability of retirement.

In terms of musculoskeletal pain (Fig. 2B), men reporting no pain in 1981 had a 14% probability of retirement within two years, in 1991 the corresponding probability was 20% (95% CI 15.1, 24.3), in 2000 it was significantly lower at 12% (95% CI 8.0, 15.3) and rose to 14% in 2010. Women reporting no musculoskeletal pain had a 21% probability of retirement in 1981; this became significantly lower in 2000 (8.4%, 95% CI 5.1, 11.7) and in 2010 (12.1%, 95% CI 8.9, 15.5). The probability of retirement for men reporting mild pain became significantly lower during the period. Severe pain among men in 1981 was associated with a 21% probability of retirement, which decreased to 13.7% in 2010. For women reporting severe pain, this fell from 31% (95% CI 23.6, 38.5) to 19% in 1991, and again to 17.6% in 2000, and to 15.4 in 2010, all of which are significantly different from 1981. The findings illustrate that physical limitations as a predictor of retirement over the time period 1981–2010 decreased in importance. In the more recent decade, people with physical limitations, especially women, stayed in employment, whereas earlier they were more likely to retire. In supplementary material Fig. S1, we present the PMs from Fig. 2 plotted over age.

## Discussion

This study explored the importance of physical functioning as a predictor for retirement over a period spanning 30 years. We investigated mobility limitations and musculoskeletal pain in the year before retirement in 1981, 1991, 2000, and 2010 for women and men in Sweden. In support of hypothesis 1, we found a trend towards physical functioning becoming less important for retirement during the study period. More specifically, results show that poor physical functioning loses its predictive value for retirement during the time period 1981 to 2010, when controlling for age, occupational-based social class, physical working conditions, and psychosocial job demands. The change is greater over time among women than men, supporting hypothesis 2. More specifically, among women, younger cohorts with limitations in physical functioning did not retire to the same extent as older cohorts with limitations. This indicates that in the more recent time period, women stayed in the labour market despite having functional limitations.

There have been several changes to the pension system in Sweden during the study period that might influence retirement decisions beyond individual health status (see details regarding the pension reforms in Supplementary material). The reforms undertaken in the 1990s introduced stronger financial incentives to delay retirement, and in addition, many exit routes out of the labour market were closed. For example, it became more difficult to qualify for disability pension, but in the period 1970–1980s one could qualify for disability pension with non-medical reasons such as long-term unemployment. Our findings suggest that these institutional changes and the economical context in each period have influenced retirement decisions, although the effect seems to vary somewhat between women and men. The cohorts with baseline data from 1981 and 1991 were not affected by these changes; however, the 2010 cohort shows a higher average retirement age and—especially among women—continued employment despite reporting problems with physical functioning. These results are consistent with König’s (2017) findings that women continue to work for financial reasons, despite strenuous working conditions or poor health.

In the year 2000, a reform on the occupational scheme for the public sector came into effect, increasing the retirement age from 63 to 65 for those born in 1938 and later. This affected occupations such as health and care workers, cleaners, and food service workers, i.e. sectors that are predominantly occupied by women. Hagen (2018) has shown that this change increased the retirement age for women by 4.5 months on average. This might partly explain why women stayed longer in the labour force despite health issues in the years 2000 and 2010 in our sample. In addition, women may be forced to stay in work for longer in order to increase their labour market income and compensate for fewer accumulated years of employment and part-time careers (Anxo et al. 2014; König 2017, 2016). We might be seeing a wave of older women on the labour market who perhaps would like to retire due to physical functioning issues, but are not able to because of institutional factors such as strong financial incentives to continue working and changes to the disability pension scheme (Coile 2015; König 2017). Research has found that having influence over one’s own work pace, work time and work intensity is important for continued work despite poor health (Dellve et al. 2016). In cases where the older workers do not have the opportunity to adjust the working environment according to their health needs, extending working life could lead to poorer health, which could further increase health inequalities.

An interesting trend is found for men with mobility limitations, where the probability of retirement increased from 1981 to 2000. This trend is consistent with the decreasing retirement age among men during these decades. Leaving the labour market through occupational schemes was common between 1990 and 2000, especially for men in non-manual occupations. The occupational scheme provided employers with a strategic way to offer older employees early retirement and was frequently used both when the public sector was cut down and reorganized in the 1990s and within the private sector. The recipients of these occupational schemes were mainly men in non-manual occupations, and the share receiving this early exit package was larger than the share retiring though disability pension (Olofsson 2002). Moreover, the partial pension scheme might have been an exit route for men having health issues, and this might partly explain the decrease in average retirement age for men in 1991 and 2000, and the following increases in retirement age when the partial pension scheme was abolished in 2001.

### Strengths and limitations

We use four waves of nationally representative data, spanning three decades. The participation rate was relatively high in all waves, ensuring generalizability to the Swedish population. In addition, we made use of a register-based outcome not liable to subjective recall error to assess retirement timing (Eyjólfsdóttir et al. 2021). Using annual register data has its limitations; one can estimate whether, in a given calendar year, income from pensions succeeded income from employment. As income from pension is generally lower than income from employment, a person must have more than six months of pension income to surmount the income from employment. This leads to the risk of determining a retirement age that is one year higher than the actual age of labour market exit for people retiring late in the calendar year. This might introduce information bias. However, a paper by Eyjólfsdóttir et al. (2021) examining different definitions of retirement age, using the same materials as the current study, did not find significant differences between self-reported retirement age and retirement age attained by this current definition, nor by using different cut-offs than 50%. Another limitation is that we employ the same definition of retirement over the three decades under study, although there have been many reforms during the period that have made the retirement process more flexible over time. Therefore, the definition might capture the timing of retirement better in some periods than in others. Moreover, retirement behaviour could be more influenced by economic context and pension reforms than by health status in specific periods, resulting in overestimation of the importance of health for retirement. An additional limitation might be that there are few respondents retiring in the two years following the interview, possibly creating statistical power issues in the interaction analysis. On the other hand, the follow-up time needs to be short for the health indicators to be relevant as predictors of retirement.

### Policy implications

As populations age, the financial sustainability of many welfare states and pension systems is threatened. In response, most European countries are implementing reforms in order to delay exit from the labour market and thus enhance fiscal sustainability (European Commission 2021). In this study, we found that the importance of physical functioning for labour market participation has declined in Sweden over a thirty-year period. These findings are promising, as physical functioning might be of less hindrance for labour force participation among older workers and thus for postponement of retirement. However, we also found that those who already have functional limitations are less likely to retire in the most recent time period, especially women. This might indicate that because of financial constraints some groups are stuck on the labour market despite poor health. In efforts to raise retirement age and increase labour force participation among older workers, policymakers should recognize the heterogeneity among older workers. Options for disability insurance and part-time retirement for older workers in adverse working conditions or for those with poor health are important in order to not enlarge health inequalities in later life.

## Conclusions

The results of this study show that the importance of physical functioning for retirement has decreased since the 1980s. The findings illustrate that in the more recent decade, people with limitations in physical functioning, especially women, stayed in employment, whereas in the earlier decades, people with limitations were more likely to retire. A risk associated with closing pathways out of the labour force for people with physical limitations and increasing financial incentives to stay in the labour market, is that such measures might amplify health inequalities in later life, and result in more demands on the social security system and the health care system.

## Supplementary Information

Below is the link to the electronic supplementary material.

Supplementary file1 (PDF 491 kb)
