# Supplementary material for: Physical functioning as a predictor of retirement: Has its importance changed over a thirty-year period in Sweden?
Source: Eur J Ageing. 2022 Sep 2;19(4):1417–28. doi: 10.1007/s10433-022-00725-y (PMC9729518; doi:10.1007/s10433-022-00725-y)
Supplement: Supplementary file 1 — Supplementary file1 (PDF 491 kb) [file 10433_2022_725_MOESM1_ESM.pdf]

Supplementary Material

Journal: European Journal of Ageing

Article title: Physical functioning as a predictor of retirement: has its importance changed over a thirty-year period in Sweden?

Harpa S. Eyjólfsdóttir[1], Neda Agahi[1], Johan Fritzell[1] and Carin Lennartsson[12]

[1] Aging Research Center, Karolinska Institutet and Stockholm University, Sweden
[2] Swedish Institute for Social Research, Stockholm University, Sweden

Contact details, corresponding author

Harpa S. Eyjólfsdóttir, PhD

Aging Research Center, Karolinska Institutet and Stockholm University

Tomtebodavägen 18 A, 171 65 Solna

Sweden

harpa.eyjolfsdottir@ki.se

ORCID: 0000-0003-1478-5550

Supplementary Material, Pension reforms

Sweden has a universal, comprehensive, tax-financed welfare system. Increasing the retirement age is seen as part of the solution for continuing to provide high-quality universal health and long-term care, and for the sustainability of the pension system. There have been four major changes to the Swedish pension system since the 1980s that are of significance to this study, and could possibly modify the importance of physical functioning for retirement.

First, a major reform to the Swedish pension system was agreed upon in the early 1990s and implemented later that decade. A notional defined contribution system (NDC) was introduced in 1994, replacing the previous pay-as-you-go system. While the ATP was based on the best 15 years of earnings during working life, the NDC calculates benefits based on earning history[1] over the entire working life and uses the average remaining life expectancy to calculate pension benefits. The NDC thus created stronger incentives for workers to delay retirement (Laun and Wallenius 2013; Sundén 2006), and was gradually applied for individuals born in 1938 and later, starting in 2001. For a detailed description of this major reform, see (Ministry of Health and Social Affairs 2009).

The second significant change was regarding the disability insurance - the dominant pathway to retirement for those who left the labour market before age 65 between 1970 and 1990 (Palme and Svensson 2010). Disability benefits are closely linked to the old-age pension system, as people shift directly to the old-age pension scheme once they reach age 65 (Stattin 2005). During the 1970s-90s, the eligibility criteria for older workers to qualify for disability pension were more lenient; in 1985, about 20% of new pensioners receiving disability benefits gave non-medical reasons such as long-term unemployment

[1] All income from employment and self-employment, and all taxable income from social insurances (such as sickness or activity benefit, parental benefit and unemployment benefit).

(Palme 2010). Since 2003, disability benefits have been granted for health reasons only. For a detailed description of the changes to the disability insurance in Sweden, see (Palme 2010).

The third major change was to the partial pension scheme that was open for employees aged 60-64 between 1976 and 2001. The scheme became very popular with a high take-up rate, especially among men. The partial pension underwent several changes, which gradually decreased the take-up rate before it was totally abolished in 2001 (Wadensjö 2006).

Finally, the introduction of “flexible retirement” in 2003 saw the statutory retirement age (65) abandoned, and people could request the old-age pension earlier (from the age of 61) as well as later (up until the age of 67) (Hagen 2013). Since 2020 the flexible retirement age has been 62-68. The eligibility age for the guarantee pension, providing a basic pension for those with no or low levels of income, has been set at 65 since 1975. Over 80% of people retire at or before the age of 65 (Pensionsmyndigheten 2020).

Supplementary Materials, Tables

Table S1. Full results from Table 3. Average marginal effects (AME) and 95% confidence intervals (CI) of retiring by *mobility limitations* for men and women in 1981, 1991, 2000 and 2010.

	Men				Women			
	1981	1991	2000	2010	1981	1991	2000	2010
	AME (95% CI)	AME (95% CI)	AME (95% CI)	AME (95% CI)	AME (95% CI)	AME (95% CI))	AME (95% CI)	AME (95% CI)
Mobility limitations								
No limitations	Reference category				Reference category			
1 limitation	-0.05 (-0.12, 0.03)	0.07 (-0.02, 0.16)	0.04 (-0.04, 0.11)	-0.01 (-0.07, 0.04)	0.04 (-0.04, 0.12)	0.07 (-0.02, 0.15)	0.03 (-0.05, 0.10)	0.05 (-0.02, 0.11)
2+ limitations	0.02 (-0.06, 0.10)	0.19 (0.06, 0.33)	0.32 (0.12, 0.52)	0.12 (0.01, 0.24)	0.20 (0.08, 0.32)	0.10 (-0.03, 0.23)	0.13 (0.03, 0.23)	0.00 (-0.08, 0.08)
Age	0.03 (0.03, 0.04)	0.04 (0.04, 0.05)	0.03 (0.03, 0.04)	0.05 (0.04, 0.05)	0.04 (0.03, 0.05)	0.03 (0.03, 0.04)	0.03 (0.02, 0.04)	0.04 (0.03, 0.05)

Table S1 continued

Occupational social class								
Unskilled manual workers	Reference category				Reference category			
Skilled manual workers	-0.02<br>(-0.09, 0.06)	0.01<br>(-0.07, 0.10)	-0.06<br>(-0.14, 0.02)	0.01<br>(-0.05, 0.08)	-0.04<br>(-0.13, 0.04)	0.00<br>(-0.07, 0.07)	-0.05<br>(-0.12, 0.01)	-0.05<br>(-0.12, 0.02)
Lower non-manuals	-0.06<br>(-0.15, 0.02)	0.04<br>(-0.07, 0.15)	0.12<br>(-0.03, 0.28)	-0.05<br>(-0.15, 0.04)	0.09<br>(-0.06, 0.24)	not estimable	-0.03<br>(-0.13, 0.07)	0.03<br>(-0.13, 0.2)
Intermediate and higher non-manuals	-0.09<br>(-0.17, -0.01)	0.01<br>(-0.08, 0.10)	-0.04<br>(-0.13, 0.05)	0.01<br>(-0.05, 0.07)	-0.04<br>(-0.12, 0.03)	0.00<br>(-0.07, 0.08)	-0.04<br>(-0.11, 0.04)	-0.02<br>(-0.09, 0.06)
Adverse physical working conditions	0.00<br>(-0.01, 0.01)	0.00<br>(-0.01, 0.01)	-0.01<br>(-0.01, 0.00)	-0.01<br>(-0.01, 0.00)	-0.02<br>(-0.03, 0.00)	0.00<br>(-0.02, 0.02)	0.00<br>(-0.01, 0.01)	0.00<br>(-0.01, 0.02)
Job demands								
Low demands	Reference category				Reference category			
Middle demands	-0.06<br>(-0.13, 0.01)	-0.04<br>(-0.12, 0.03)	0.00<br>(-0.06, 0.05)	0.02<br>(-0.03, 0.08)	-0.08<br>(-0.15, -0.01)	-0.09<br>(-0.17, -0.01)	-0.05<br>(-0.12, 0.01)	-0.13<br>(-0.21, -0.06)
High demands	-0.07<br>(-0.14, 0.00)	-0.02<br>(-0.09, 0.05)	-0.03<br>(-0.08, 0.02)	0.02<br>(-0.03, 0.08)	-0.05<br>(-0.14, 0.04)	-0.08<br>(-0.17, 0.01)	-0.09<br>(-0.16, -0.02)	-0.13<br>(-0.21, -0.05)

Table S2. Full results from Table 3. Average marginal effects (AME) and 95% confidence intervals (CI) of retiring by *musculoskeletal pain* for men and women in 1981, 1991, 2000 and 2010.

	Men				Women			
	1981	1991	2000	2010	1981	1991	2000	2010
	AME (95% CI)	AME (95% CI)	AME (95% CI)	AME (95% CI)	AME (95% CI)	AME (95% CI))	AME (95% CI)	AME (95% CI)
Musculoskeletal pain								
No pain	Reference category				Reference category			
Mild pain	0.02 (-0.04, 0.09)	-0.05 (-0.11, 0.02)	-0.01 (-0.06, 0.04)	-0.05 (0.06, -0.09)	-0.06 (-0.12, 0.01)	-0.07 (-0.14, -0.01)	0.03 (-0.03, 0.08)	0.00 (-0.05, 0.05)
Severe pain	0.07 (0.00, 0.14)	0.02 (-0.06, 0.10)	0.05 (-0.01, 0.12)	0.00 (0.89, -0.06)	0.10 (0.02, 0.19)	0.02 (-0.05, 0.09)	0.03 (0.03, 0.15)	0.05 (-0.02, 0.11)
Age	0.03 (0.03, 0.04)	0.04 (0.04, 0.05)	0.04 (0.03, 0.04)	0.05 (0.00, 0.04)	0.04 (0.04, 0.05)	0.04 (0.03, 0.04)	0.00 (0.02, 0.04)	0.04 (0.03, 0.05)

Table S2 continued

Occupational social class								
Unskilled manual workers	Reference category				Reference category			
Skilled manual workers	-0.02<br>(-0.10, 0.06)	0.02<br>(-0.07, 0.11)	-0.07<br>(-0.15, 0.01)	0.01<br>(0.75, -0.05)	-0.03<br>(-0.12, 0.05)	0.01<br>(-0.06, 0.08)	0.04<br>(-0.13, 0.02)	-0.04<br>(-0.11, 0.02)
Lower non-manuals	-0.07<br>(-0.15, 0.02)	0.04<br>(-0.08, 0.16)	0.12<br>(-0.05, 0.30)	-0.07<br>(0.19, -0.17)	0.09<br>(-0.05, 0.24)	not estimable	0.05<br>(-0.17, 0.03)	0.04<br>(-0.13, 0.20)
Intermediate and higher non-manuals	-0.09<br>(-0.18, 0.00)	0.00<br>(-0.10, 0.10)	-0.04<br>(-0.13, 0.05)	0.02<br>(0.59, -0.04)	-0.06<br>(-0.13, 0.02)	0.00<br>(-0.07, 0.08)	0.04<br>(-0.13, 0.03)	-0.01<br>(-0.09, 0.06)
Adverse physical working conditions	0.00<br>(-0.01, 0.01)	0.00<br>(-0.01, 0.01)	0.00<br>(-0.01, 0.00)	0.00<br>(0.17, -0.01)	-0.02<br>(-0.03, 0.00)	0.00<br>(-0.02, 0.02)	0.01<br>(-0.01, 0.01)	0.00<br>(-0.01, 0.02)
Job demands								
Low demands	Reference category				Reference category			
Middle demands	-0.06<br>(-0.13, 0.01)	-0.03<br>(-0.1, 0.04)	-0.01<br>(-0.07, 0.04)	0.01<br>(0.64, -0.04)	-0.08<br>(-0.16, -0.01)	-0.10<br>(-0.18, -0.01)	0.04<br>(-0.14, 0.01)	-0.13<br>(-0.21, -0.06)
High demands	-0.08<br>(-0.15, -0.01)	-0.02<br>(-0.10, 0.05)	-0.03<br>(-0.09, 0.03)	0.01<br>(0.75, -0.05)	-0.04<br>(-0.13, 0.05)	-0.10<br>(-0.19, -0.01)	0.04<br>(-0.18, -0.02)	-0.13<br>(-0.21, -0.05)

Supplementary material, Fig S1

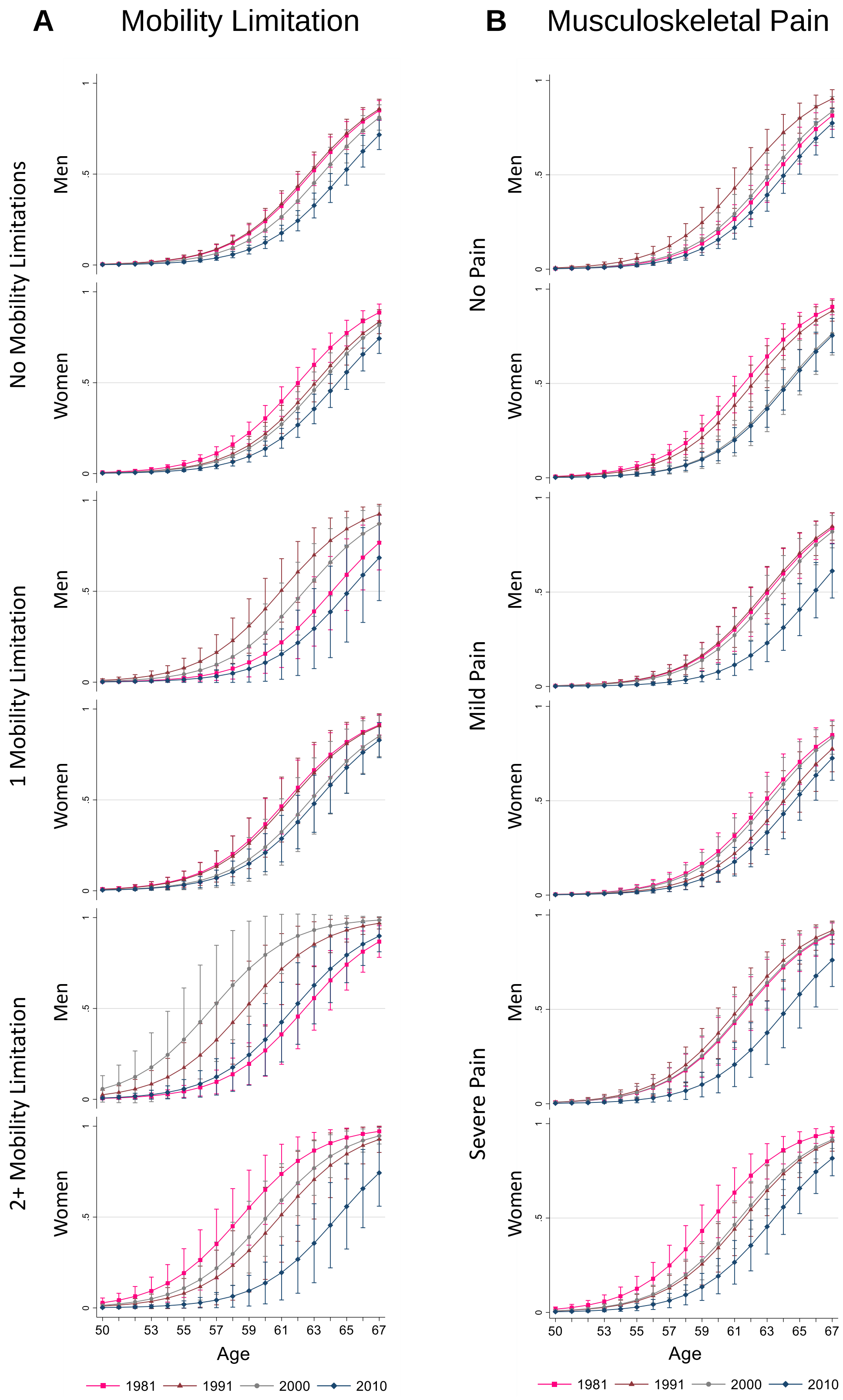

Fig. S1 Predictive margins (PM) and 95% Confidence Intervals (CI) of mobility limitations (A) and musculoskeletal pain (B) for men and women on the probability of retirement within two years plotted over age, including a three-way interaction between gender, period, and the respective health outcome, while adjusting for age, occupational-based social class, adverse physical working conditions, and job demands

In **Fig. S1** we present the predictive margins from **Fig. 2** plotted over age. **Fig. S1 part A** shows the PM of retirement within two years over age by mobility limitations for men and women. A horizontal line at 0.5 PM shows when the probability of retirement within two years reaches 50%; for example, a man with no mobility limitations reaches 50% probability of retiring at age 63 in 1981 and 1991, at age 64 in 2000, and at age 65 in 2010. Among men and women with no mobility limitations, we see a trend towards delayed retirement over the period. Among men who had one mobility limitation, we see higher probability of retirement in 1991 and 2000 compared to 1981 and 2010. When looking at men and women having two or more mobility limitations, we see more variation over age. In the year 2000, men reached 50% probability of retirement within two years at age 57, and at age 59 in 1991. This is a substantial change from reaching the same probability at age 62.5 in 1981 and 2010. Women who had two or more mobility limitations also show large variation over the time period, but unlike men, the highest probability of retirement is found in 1981, followed by 1991 and 2000. The 2010 period stands out for much lower probability of retirement, only passing the 50% mark at age 64.5.

Part B of Fig. S1 shows the results regarding musculoskeletal pain. The variation between periods is not large, but throughout all states of pain and for both men and women we see lower probabilities of retirement in the year 2010 compared to the earlier periods. Especially we see lower probabilities for retirement for men experiencing mild and severe pain in 2010 as compared to earlier years; men experiencing mild pain in 2010 pass the 50% mark at age 66 but earlier at age 63. Men experiencing severe pain in 2010 pass the 50% mark at age 64.5, but earlier at age 62. We see the same trend for women; in 1981, they passed the 50% threshold for retirement at age 60 if they were experiencing severe pain, this rose to age 63.5 in 2010.

Supplementary Material, References

Hagen J (2013) *A History of the Swedish Pension System*. *Uppsala Center for Fiscal Studies. Department of Economics*. Uppsala University. Retrieved from http://www.nek.uu.se/Pdf/20137ucfs.pdf

Laun T and J Wallenius (2013) A life cycle model of health and retirement: The case of Swedish pension reform. *Journal of Public Economics 127*, 127–136. https://doi.org/10.1016/j.jpubeco.2013.11.002

Ministry of Health and Social Affairs (2009) *The Swedish Pension Agreement and Pension Reform*. Fritzes, Stockholm. Retrieved from http://www.government.se/contentassets/3d321fd499da48928de201abe43a558b/the-swedish-pension-agreement-and-pension-reform-ds-200953

Palme M and I Svensson (2010) Incentives to Retire, the Employment of the Old, and the Employment of the Young in Sweden. *Social Security Programs and Retirement around the World: The Relationship to Youth Employment* (February), 295–318.

Pensionsmyndigheten (2020) *Pensionsåldrar och arbetslivets längd [Retirement ages and length of working life]*.

Stattin M (2005) Retirement on grounds of ill health. *Occupational and Environmental Medicine 62*(2), 135–140. https://doi.org/10.1136/oem.2004.015669

Sundén A (2006) The Swedish Experience with Pension Reform. *Oxford Review of Economic Policy 22*(1), 133–148. https://doi.org/10.1093/oxrep/grj009

Wadensjö E (2006) *Part-time pensions and part-time work in Sweden* (No. 2273). *IZA Discussion Paper*. Bonn, Germany.
